# The immune factors involved in the rapid clearance of bacteria from the midgut of the tick *Ixodes ricinus*

**DOI:** 10.3389/fcimb.2024.1450353

**Published:** 2024-08-13

**Authors:** Melina Garcia Guizzo, Helena Frantová, Stephen Lu, Tereza Kozelková, Kristýna Číhalová, Filip Dyčka, Alena Hrbatová, Miray Tonk-Rügen, Jan Perner, José M. Ribeiro, Andrea C. Fogaça, Ludek Zurek, Petr Kopáček

**Affiliations:** ^1^ Institute of Parasitology, Biology Centre of the Czech Academy of Sciences, České Budějovice, Czechia; ^2^ Vector Biology Section, Laboratory of Malaria and Vector Research, National Institute of Allergy and Infectious Diseases (NIAID), Bethesda, MD, United States; ^3^ Faculty of Science, University of South Bohemia, Ceske Budejovice, Czechia; ^4^ Department of Microbiology, Nutrition and Dietetics/CINeZ, Czech University of Life Sciences, Prague, Czechia; ^5^ Central European Institute of Technology (CEITEC), University of Veterinary Sciences, Brno, Czechia; ^6^ Institute for Insect Biotechnology, Justus Liebig University of Giessen, Giessen, Germany; ^7^ Department of Parasitology, Institute of Biomedical Sciences, University of São Paulo, São Paulo, Brazil

**Keywords:** tick, Ixodes, midgut microbiome, immune system, antimicrobial peptide, defensin, *Micrococcus luteus*

## Abstract

Ticks are obligate hematophagous arthropods that transmit a wide range of pathogens to humans as well as wild and domestic animals. They also harbor a non-pathogenic microbiota, although our previous study has shown that the diverse bacterial microbiome in the midgut of *Ixodes ricinus* is quantitatively poor and lacks a core. In artificial infections by capillary feeding of ticks with two model bacteria (Gram-positive *Micrococcus luteus* and Gram-negative *Pantoea* sp.), rapid clearance of these microbes from the midgut was observed, indicating the presence of active immune mechanisms in this organ. In the current study, RNA-seq analysis was performed on the midgut of *I. ricinus* females inoculated with either *M. luteus* or *Pantoea* sp. or with sterile water as a control. While no immune-related transcripts were upregulated by microbial inoculation compared to that of the sterile control, capillary feeding itself triggered dramatic transcriptional changes in the tick midgut. Manual curation of the transcriptome from the midgut of unfed *I. ricinus* females, complemented by the proteomic analysis, revealed the presence of several constitutively expressed putative antimicrobial peptides (AMPs) that are independent of microbial stimulation and are referred to here as ‘guard’ AMPs. These included two types of midgut-specific defensins, two different domesticated amidase effector 2 (Dae2), microplusin/ricinusin-related molecules, two lysozymes, and two gamma interferon-inducible lysosomal thiol reductases (GILTs). The *in vitro* antimicrobial activity assays of two synthetic mature defensins, defensin 1 and defensin 8, confirmed their specificity against Gram-positive bacteria showing exceptional potency to inhibit the growth of *M. luteus* at nanomolar concentrations. The antimicrobial activity of midgut defensins is likely part of a multicomponent system responsible for the rapid clearance of bacteria in the tick midgut. Further studies are needed to evaluate the role of other identified ‘guard’ AMPs in controlling microorganisms entering the tick midgut.

## Highlights

- The capillary feeding triggers dramatic transcriptomic changes in the midgut of unfed *Ixodes ricinus* females.
- Bacterial inoculation by capillary feeding does not induce upregulation of immune-related factors in the *I. ricinus* midgut.
- The immune-related factors are constitutively present in the midgut of unfed ticks and referred to as ‘guard’ AMPs,
- Midgut-specific defensins exert a strong antimicrobial activity against Gram-positive bacteria.

## Introduction

The commensal gut microbiota may contribute to many aspects of a host biology including food digestion, nutrient production, defense against pathogens, and detoxification of harmful substances (Azambuja et al., 2005; Mcfall-Ngai et al., 2013). Arthropods are the largest phylum of all living organisms and exhibit a wide range of physiological dependence on the gut microbiome. In blood-feeding arthropods, the gut microbiome is usually abundant and stable and plays a critical role in vector physiology and vector competence (Azambuja et al., 2005; Caragata and Short, 2022; Pavanelo et al., 2023; Wang et al., 2023). For example, the gut microbiota of *Aedes aegypti* is required for normal fecundity and vector competence (Harrison et al., 2023) and *Rhodnius prolixus* has been shown to rely on gut microbes to reach adulthood (Gilliland et al., 2023). In contrast, ticks appear to be an exception. A poor and unstable bacterial microbiome in the midgut has been described for the genera *Ixodes*, *Amblyomma*, and *Rhipicephalus*, indicating that this trait is shared by all tick species (Ross et al., 2018; Guizzo et al., 2020; Pavanelo et al., 2020; Maldonado-Ruiz et al., 2021).

With the aim of manipulating the microbial abundance of the tick midgut, we previously artificially fed *Ixodes ricinus* females with Gram-positive (*Micrococcus luteus*) and Gram-negative bacteria (*Pantoea* sp.) isolated from the unfed female midgut. The organ could not be colonized as the bacteria were eliminated very quickly (Guizzo et al., 2022). This led us to hypothesize that bacterial clearance triggered in the midgut of unfed females is critical for maintaining the low microbial levels during blood feeding.

**Figure 1:**
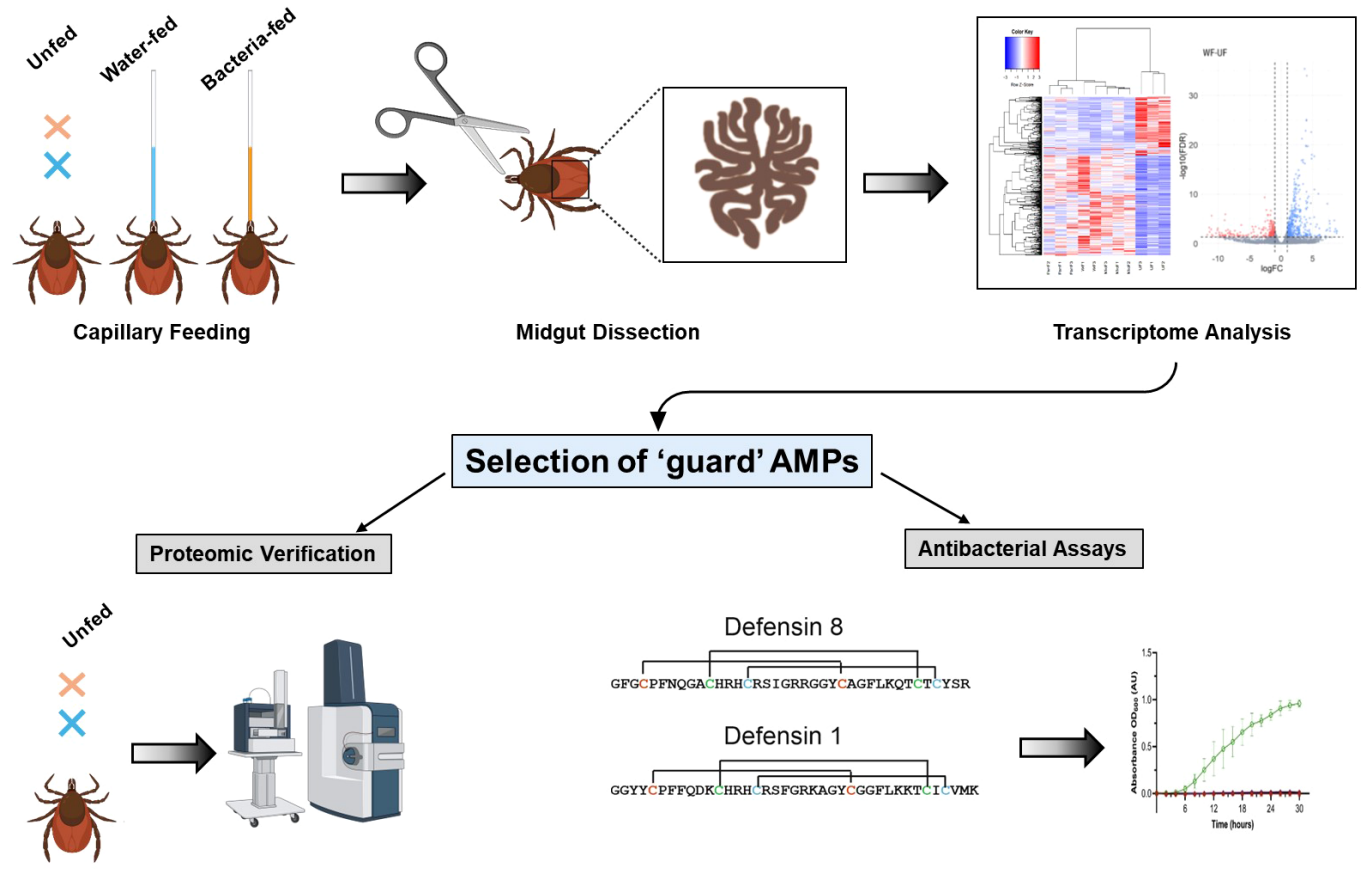
Workflow of the experiments conducted in this study. The midgut of ticks inoculated with bacteria or uninfected (feeding sterile water) and unfed ticks were dissected and submitted for RNA-seq analysis. Identification of transcripts encoding potential antimicrobial ‘guard’ peptides constitutively expressed in unfed ticks was verified by the proteomic analysis. The antimicrobial activity of two midgut-specific synthetic mature defensins was tested *in vitro* against selected Gram-positive and Gram-negative bacteria.

To decipher the factors responsible for controlling bacterial proliferation in the midgut of *I. ricinus* females, in the current study, schematically outlined in the **Figure 1**, we determined the differential expression of transcripts in response to inoculation with *M. luteus* or *Pantoea* sp. While the expression of immune transcripts was apparently not upregulated upon bacterial feeding, transcripts such as defensins, domesticated amidase effector 2 (dae2), and microplusin (ricinusin) were found to be highly expressed in the midgut of unfed females. We propose that these antimicrobial peptides (AMPs) act as guards of the tick midgut and contribute to control of invading microorganisms.

## Materials and methods

### Biological materials

Adult *I. ricinus* females were collected with a flag in a forest near České Budějovice, Czech Republic. Adult females were kept in humid chambers at 24°C, a relative humidity of ~95% and a day/night period of 15/9 hrs. and blood-fed on guinea pigs (*Cavia porcellus*). A pathogen-free laboratory colony of *I. ricinus* ticks was established from the laid eggs and underwent the entire developmental cycle (larvae, nymphs, adults) in the tick breeding facility of the Institute of Parasitology, Biology Centre CAS. All experimental animals were handled in accordance with the Animal Protection Law of the Czech Republic No. 246/1992 Sb., ethics approval No. 25/2020.

### Capillary inoculation of ticks with bacteria

Unfed adult *I. ricinus* females from the pathogen-free laboratory colony described above were experimentally orally inoculated using the glass capillary with either *Pantoea* sp. (10^7^ CFU/ml) or *M. luteus* (10^7^ CFU/ml) previously isolated from the unfed midgut of *I. ricinus* females (Guizzo et al., 2022). As a control, the ticks were fed autoclaved tap water (uninoculated ticks). All ticks were fed for 1 hour at 28°C in a humid chamber. The volume ingested by each tick was determined using a ruler and converted into the number of bacterial cells ingested per tick. A control group of unfed females was also included. Prior to dissection, ticks were washed with 0.05% sodium hypochlorite for 1 min, followed by 1 min in 70% ethanol and 3 consecutive washes in sterile water for 10 seconds each to prevent microbial contamination from the tick surface (Binetruy et al., 2019). Ticks were dissected and the total RNA was isolated from pools of 8 midguts per replicate using the NucleoSpin^®^ RNA kit (Macherey-Nagel, USA) and eluted in 40 µl of RNAse-free water. The isolated RNA was quantified using a NanoDrop^®^ND-1000 spectrophotometer (Thermo Scientific, USA), and three independent biological replicates from each group were submitted for Illumina sequencing. Quality control of submitted RNA samples, construction of non-stranded cDNA libraries and Illumina reading of 150 bp paired ends were performed by Novagene Co., Ltd. as previously described in detail for the red poultry-mite RNAseq analysis (Ribeiro et al., 2023).

### Transcriptome analysis

The quality of the raw Illumina reads obtained from the midgut of unfed, uninoculated, and inoculated *I. ricinus* females were inspected using the FastQC tool (https://www.bioinformatics.babraham.ac.uk/projects/fastqc). Illumina adaptors and low-quality sequences, specifically those with a Phred quality score (Q) below 20, were removed using the TrimGalore software (https://github.com/FelixKrueger/TrimGalore). Subsequently, the clean reads from all libraries were merged and subjected to *de novo* assembly using Trinity (2.9.0) (Grabherr et al., 2011) and ABySS (2.3.1) (Simpson et al., 2009). Sequences obtained from both assemblers were merged and the sequences that were at least 95% identical were consolidated using the CD-HIT software (Fu et al., 2012). Putative coding sequences (CDSs) were extracted based on the presence of a putative signal peptide or similarity to previously deposited sequences. All potential open reading frames (ORFs) of at least 175 nucleotides were extracted and blasted against several databases, including a subset of the non-redundant protein database from NCBI(NR), UNIPROTKB, refseq-vertebrate and a tick-specific database (TSF) (Ribeiro and Mans, 2020). CDSs were extracted if they had a coverage of 70% or more for a matching protein. In parallel, all ORFs starting with a methionine and at least 40 amino acids long were submitted to the signalP (3.0) software (Bendtsen et al., 2004). Sequences containing a putative signal peptide were assigned to ORFs, and the methionine codon closest to the 5’ terminal was selected as the start codon of the transcript. The relative quantification of each CDS was estimated using the transcript per million (TPM) parameter by mapping the trimmed Illumina reads to the final list of CDSs using RSEM software (Li and Dewey, 2011). To assess the quality of our *de novo* assembly and the CDS extraction pipeline, we used the benchmark of the universal single-copy ortholog (BUSCO) with the Arachnida database (Seppey et al., 2019). For functional annotation and differential expression analysis, we extracted CDSs with an average TPM of at least 5 in one of the biological conditions (unfed, uninfected or infected). The CDSs were blasted against multiple databases (subset of the NR database, UNIPROTKB, Enzyme Committee (EC), MEROPS, CDD, KOG, PFAM, SMART, and TSF) and assigned to specific functional classes, including their coverage and identity scores, using an *in-house* program that scans a vocabulary (~400 words) and the order of occurrence in the proteins matched from the BLAST results. The annotated CDSs were exported to a Windows-compatible, hyperlinked Excel file, available for download (see Data Availability below, **Supplementary File 1**).

### Proteomic analysis of midguts from unfed *I. ricinus* females

Midguts of unfed *I. ricinus* females were dissected on a drop of ice-cold phosphate-buffered saline (PBS) and transferred to a microtube and stored at −80°C until further use. Proteomic analysis was performed in triplicates, using 10 midguts per sample. Samples were homogenized in 200 μL of 50 mM Na-phosphate buffer, pH 7.5, supplemented with 7 M urea, 2 M thiourea, 2% CHAPS and Halt™ protease inhibitors (Thermo Fisher Scientific) and then 10 µg of proteins were digested in solution with trypsin (Pierce Trypsin Protease, MS Grade, Thermo Fisher Scientific) as described previously (Kozelkova et al., 2023). After desalting using the Stage tips solid phase C18 disc (Empore), peptides were subjected to NanoLS ESI/MS/MS analysis performed on an UltiMate™ 3000 RSLCnano system (Thermo Fisher Scientific) coupled online to the timsTOF Pro mass spectrometer (Bruker Daltonics) (Kozelkova et al., 2023). The data were analyzed using the MaxQuant software (version 1.6.14) with the integrated search engine Andromeda (Cox and Mann, 2008). The databases of *I. ricinus* and the guinea pig (08. 06. 2021 and 28.07.2021, respectively) available in the Uniprot and the contaminant database included in the MaxQuant software were used for protein identification. Data analysis parameters and label-free quantification (LFQ) algorithms were applied as described by (Kozelkova et al., 2023). LFQ intensity values were log2 transformed in Perseus software (version 1.6.14.0 (Tyanova et al., 2016)). Contaminants, host proteins, and proteins identified in only one of the three replicates were excluded from further analysis.

### Defensin sequences and synthetic peptides

Defensin 1 [corresponding to the prepro-defensin 1 according to the recently published annotation of the *I. ricinus* genome (Cerqueira De Araujo et al., 2024)] was previously identified as encoded by the transcript Ir-113775, which is upregulated in the later phase of tick feeding, as revealed by a transcriptomic analysis of *I. ricinus* midgut (Perner et al., 2016). The sequence of the mature defensin 1 is GGYYCPFFQDKCHRHCRSFGRKAGYCGGFLKKTCICVMK (Mw 4.491 Da). The three predicted disulfide bridges connect the cysteine residues C5-C26, C12-C34 and C16-C36. The defensin encoded by the transcript seqSigP-776440 in the midgut transcriptome of unfed *I. ricinus* females (current study) corresponds to prepro-defensin 8 (Cerqueira De Araujo et al., 2024). The sequence of the mature defensin 8 is GFGCPFNQGACHRHCRSIGRRGGYCAGFLKQTCTCYSR (Mw 4.194 Da) with a disulfide bridge pattern linking C4-C24, C11-C32 and C15-C34. Mature defensins 1 and 8 with targeted cross-linking of cysteine residues were synthesized, purified and analyzed by HPLC and mass spectrometry by Pepmic Co., Ltd (Suzhou, China) with a declared purity >90% and delivered as a lyophilized powder.

### Antibacterial assays

*Staphylococcus aureus* (CCM 4223), *Staphylococcus epidermidis* (CCM 7221), *Pseudomonas aeruginosa* (CCM 3955), and *Escherichia coli* (D31) were obtained from the Czech Collection of Microorganisms, Faculty of Science, Masaryk University in Brno, Czech Republic. *Micrococcus luteus* and *Microbacterium maritypicum* were isolated from the midgut of *I. ricinus* (Guizzo et al., 2022) on Tryptic Soy Agar (TSA) (Sigma Aldrich, USA) and stored at -80°C in the cryo-medium. The bacterial strains were cultured on TSA at 37°C overnight, diluted in Mueller Hinton (MH) broth (Sigma Aldrich, USA) to OD_600_ = 0.1 AU, and then diluted 100× to reach the cell density 1–2 × 10^6^ CFU/mL.

One-millimolar stock solutions of the synthetic mature defensins 1 and 8 were prepared in sterile distilled water. Assays were performed as previously described (Tonk et al., 2015). Briefly, 10 μL of the peptide stock solutions were serially diluted 2-fold in MH medium to which bacteria suspensions adjusted to an initial OD_600_ = 0.005 or OD_600_ = 0.05 for *M. luteus* were added. The concentrations of synthetic defensins tested ranged from 250 μM to 8 nM. Bacterial growth was monitored at 30°C by reading the OD_600_ every 20 min for 48 h (orbital shaking) on the Eon™ Microplate Spectrophotometer (BioTek Instruments, VT, USA). As a positive control, bacteria were cultured without defensins. The measured OD_600_ of the negative control (MH medium alone) was subtracted from the absorbance values as a background. All measurements were performed in independent triplicates.

### Effect of the reduction of the disulfide bridges of defensins on the growth inhibition of *M. luteus*

Ten microliters of 1 mM solutions of the synthetic mature defensin 1 or 8 were mixed with 10 μL of 20 mM dithiothreitol (DTT) and heated to 75°C. After 5 min, the solutions were spun down, diluted 5-fold in MH medium (to a final concentration of 100 μM for peptides and 2 mM for DTT) and used as stock solutions for the antibacterial assay. Untreated peptides, DTT-treated peptides or DTT only were serially diluted 2-fold in MH medium to which a suspension of *M. luteus* (OD_600_ = 0.05) was added. The peptide concentrations tested ranged from 25 μM to ~ 0.8 nM and from 500 μM to 15 nM for DTT alone. Bacteria were incubated at 30°C and the growth was measured discontinuously from 24 h to 64 h using a Tecan M200 Infinite Pro Microplate Reader (Tecan, Austria).

### Statistical analysis

Differential expression analysis of a putative CDS was performed using the edgeR package (Robinson et al., 2010) for R. The expression of a CDS was considered statistically different if a log2(fold change) was greater than 1 or less than -1 and the false discovery rate (FDR) was less than 0.05. The volcano plot was generated using the *ggplot2* package for R.

### Data availability

The transcriptome data were deposited to the National Center of Biotechnology and Information (NCBI) under the BioProject PRJNA685402, with the BioSamples SAMN17086831 - SAMN17086834. The raw Illumina reads were submitted to the Sequence Reads Archives (SRA) under accessions SRR13257934 – SRR13257945. The putative CDS were submitted to the Transcriptome Shotgun Assembly (TSA) under the accession GIYG00000000. The **Supplementary File 1** can be downloaded from the following link: https://proj-bip-prod-publicread.s3.amazonaws.com/transcriptome/Iricinus/InfectedMg/Supplementary_file_1.zip. The mass spectrometry proteomics data from tick hemolymph were deposited to the ProteomeXchange Consortium via the PRIDE partner repository PXD053128.

## Results

### Overview of the transcriptome data of the midgut of *I. ricinus*

Illumina sequencing of 12 libraries from the midgut of unfed, uninoculated (water fed), and inoculated *I. ricinus* yielded a total of 461,359,915 high-quality reads. These reads were assembled *de novo*, resulting in 679,580 putative transcripts. Alignment of the trimmed Illumina reads to these transcripts yielded consistent alignment rates across all libraries, averaging 29.2% ± 1.1%, with the exception of one water fed tick (sample WF2), which had a significantly lower mapping rate of 8.86% and was excluded from further analysis. The putative CDSs were extracted with a minimum TPM value of 5 in at least one biological condition, resulting in a final set of 17,185 putative CDS (**Supplementary File 1**). The quality of *de novo* assembly and CDS prediction by BUSCO showed that our current dataset has a high overall quality with a completeness of 73.1%, which is consistent with previous RNA-seq analyses of the tick midgut (Lu et al., 2023, 2024).

**Figure 2:**
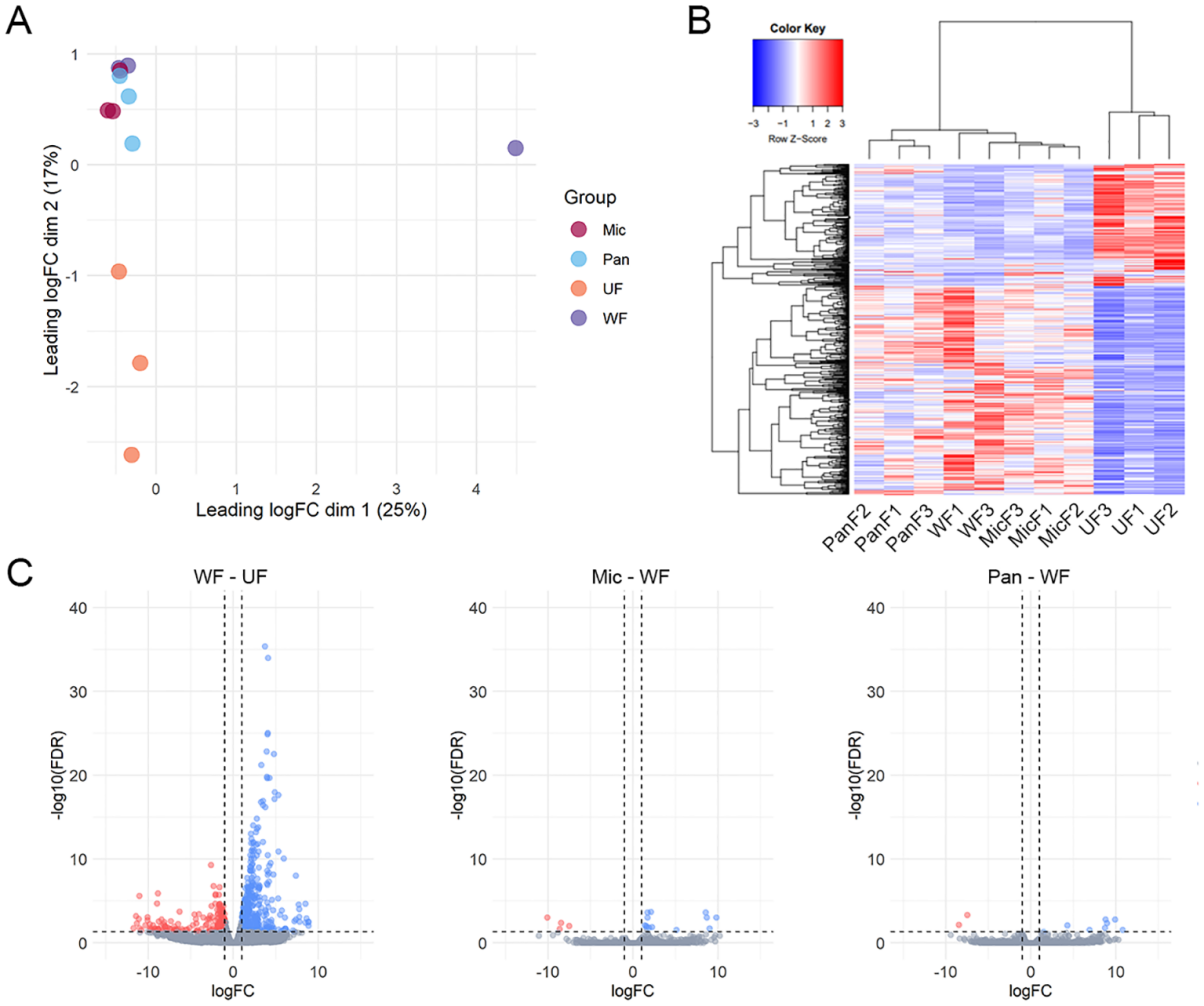
Overview of *I. ricinus* midgut RNA-sequencing. **(A)** Multidimensional plot based on the transcripts that presented an average TPM value ≥ 5 in at least one biological condition, unfed (UF) or artificially fed with water (WF), *Pantoea* (Pan) or *M. luteus* (Mic). **(B)** Heatmap plot using the normalized TPM values of each transcript. PanF1,2,3 – *Pantoea* sp. – fed (inoculated); WF1,3 – water fed (uninoculated); MicF1,2,3 – *M. luteus* fed (inoculated); UF1,2,3 – unfed. **(C)** Volcano plots highlighting the differentially expression transcripts identified between the pairwise comparisons between uninoculated (water fed, WF) and unfed (UF), *M. luteus*-inoculated ticks (Mic) and WF or *Pantoea*-inoculated ticks and WF. Transcripts were considered differentially expressed when a LogFC (FC – fold change) higher than 1 or lesser than -1 (vertical line) alongside an FDR (False Discovery Rate) < 0.05 (horizontal line) was found. Upregulated transcripts are shown as blue dots, downregulated transcripts as red dots and non-differentially expressed transcripts as gray dots.

Initial data exploration through dimensional analysis revealed that all biological samples clustered well within their respective biological groups, except for uninoculated (water fed, WF) sample WF2 (**Figure 2A**). This outlier in combination with the strikingly low mapping rates indicates a significant sequencing bias in this sample. Therefore, we decided to exclude this sample from our study. Furthermore, our dimensional analysis revealed two prominent clusters that distinguished between unfed and fed ticks. Remarkably, despite the exposure to Gram-positive and Gram-negative bacteria, the transcriptional profile of inoculated ticks strongly resembled that of the uninoculated (WF) ticks.

Hierarchical clustering of the 17,185 putative transcripts illustrated the congruence among our biological replicates and the transcriptional coherence within our artificially fed samples (**Figure 2B**). In particular, a biphasic pattern emerged between fed and unfed ticks, with transcripts that were abundant in unfed ticks being downregulated in fed samples and *vice versa*. This pattern emphasizes the onset of feeding as the primary stimulus for transcriptional changes in the tick midgut and confirms the success of capillary feeding as a method to artificially infect ticks.

**Figure 3:**
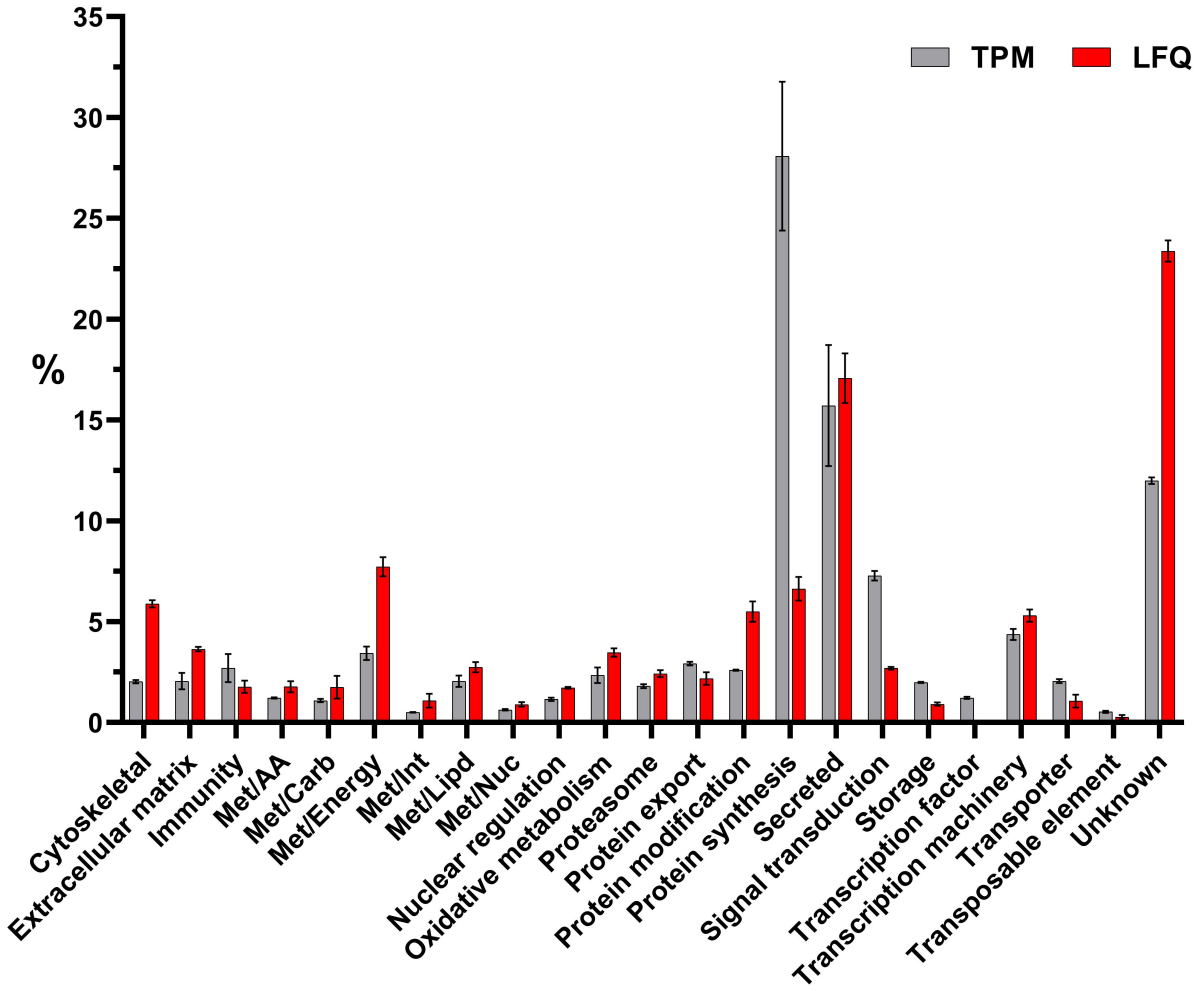
Functional classification of transcripts (in gray) or proteins (in red) identified in the midguts of unfed *I. ricinus* females by transcriptomic and proteomic analysis, respectively. Bars represent the average TPM (transcript per million) or LFQ (label-free quantification) percentage values for each class, while error bars represent the standard deviation of the mean.

Furthermore, the differential expression analysis revealed 683 modulated CDSs between uninoculated (WF) and unfed (UF) ticks (**Figure 2C**). In contrast, we observed a limited number of CDSs differentially expressed between *M. luteus* (Mic) and uninoculated (WF) ticks (17 CDSs) or between *Pantoea* sp. (Pan) and uninoculated (WF) ticks (10 CDSs). Notably, none of the 27 modulated CDSs between bacteria inoculated and uninoculated ticks (WF) were included in the Immunity class (**Supplementary File 1**). However, we observed several CDSs of antimicrobial peptides in the midgut of unfed ticks (**Supplementary File 1**). In total, these CDSs accounted for 2.7% of the total (**Figure 3**).

### Potential antimicrobial ‘guard’ peptides in the midgut of unfed females

The rapid clearance of bacteria from the midgut of *I. ricinus* (Guizzo et al., 2022) combined with the results of differential transcriptome analysis prompted us to search the midgut of unfed females for transcripts encoding proteins and peptides known in the literature to exert antimicrobial (bacteriolytic or bacteriostatic) activity in ticks (Fogaça et al., 2021). These included the small (~ 4 kDa) cationic peptides defensins ( (Wu et al., 2022)), Dae-2 (Chou et al., 2015; Hayes et al., 2020), cysteine- and histidine-rich microplusins (ricinusin) (Fogaca et al., 2004; Lai et al., 2004), and c-type lysozymes (Kopacek et al., 1999; Simser et al., 2004; Tanaka et al., 2010). In addition to these well-characterized molecules, we also found the homologue of a glycine-rich acanthoscurrin from the tarantula spider that has been reported to be active against *E. coli* (Gram-negative) and the yeast *Candida albicans* (Lorenzini et al., 2003). Finally, we turned our attention to the putative gamma-interferon-inducible lysosomal thiol reductase (GILT), which is known to exert a function in MHC class II-restricted antigen processing in mammals (West and Cresswell, 2013) and has been shown to be involved in innate immunity in *Drosophila* and the malaria mosquito vector *Anopheles gambiae* (Kongton et al., 2014; Schleicher et al., 2018; Yang et al., 2020). These transcripts were reproducibly and consistently present in the midgut of unfed females (**Table 1**), with TPM levels unaffected by bacterial or sterile water capillary feeding (**Supplementary File 1**).

**Table 1:** Antimicrobial peptides in the unfed female midgut of *Ixodes ricinus* identified by transcriptomic and proteomic analysis.

Transcript/protein name	Transcript ID	GenBank Access. No.	UF1 TPM	UF2 TPM	UF3 TPM	Protein-Uniprot Access. No.	Group 1 Log2 LFQ	Group2 Log2 LFQ	Group 3 Log2 LFQ
**Defensin 8**	seqSigP-776440	MBK3725883.1	2332,86	4663,32	195,98	V5IGJ0	17.99	18.95	14.45
**Defensin 1**	nd	JAP75178.1	-	-	-	Q7YXK5	19.23	19.69	18.04
**Dae2-type1**	Irseq_658490	MBK3726561.1	4197,98	4682,16	2256,61	A0A131Y7K5	15.27	15.78	14.86
**Dae2-type2**	Irseq_1462568	MBK3727421.1	148,94	48,27	16,71	nd	-	-	-
**Microplusin/Ricinusin**	Irseq_818892	MBK3728589.1	557,27	267,62	517,72	A0A6B0UTF6	18.26	18.23	17.18
**Microplusin - type 2**	Irseq_1035813	MBK3730421.1	21,51	41,08	77,71	nd	-	-	-
**c-type lysozyme 1**	seqSigP-924189	MBK3722801.1	476,02	1616,31	393,08	nd	-	-	-
**Lysozyme-like**	Irseq_1030185	MBK3725204.1	7,71	20,79	1,67	nd	-	-	-
**Acanthoscurrin-like**	seqSigP-1078729	MBK3720576.1	28,89	13,84	37,66	nd	-	-	-
**GILT**	Irseq_909409	MBK3726697.1	812,9	658,04	435,1	A0A0K8RMQ6	15.83	15.01	14.03
**GILT-like**	seqSigP-678986	MBK3724106.1	883,2	1035,0	1088,6	V5HBD3	15.44	15.05	14.92

nd, not detected.

To confirm the presence of the potential ‘guard’ AMPs in the unfed midgut of females and to shed the light on other proteins that may serve as ‘guard’ defense molecules against ingested environmental bacteria, a semiquantitative label-free proteomic analysis was performed. A total of 920 proteins were identified in three independent biological replicates. After filtering step, a total of 689 proteins (host and ticks) remained, among which the 650 *I. ricinus*–specific proteins were used for further analysis (**Supplementary Excel Table S2**). From the 10 CDSs of ‘guard’ AMPs identified in the midgut of unfed females, the presence of 5 was confirmed by proteomic analysis (**Table 1**). In addition to the RNA-seq dataset, proteomic analysis revealed the presence of another form of defensin, named defensin 1 (**Table 1**), which has previously been found to be expressed in the midgut of *I. ricinus* during the later stages of feeding (Perner et al., 2016).

### Gene expression of potential ‘guard’ antimicrobial peptides

The transcript seqSigP-776440, which encodes defensin 8 [according to the recent annotation of the *I. ricinus* genome (Cerqueira De Araujo et al., 2024)], was among the most abundantly expressed immune CDSs in the midgut of unfed *I. ricinus* (**Supplementary File 1**). In addition, its encoded protein (V5IGJ0) was detected in the midgut of all groups of ticks examined (**Table 1**). Although no transcript encoding defensin 1 [Ir-113775 (GenBank JAP75178) (Perner et al., 2016)] was identified, the abundance of its encoded protein Q7YXK5 was relatively high in the midgut of unfed females (**Table 1**). The sequences of *I. ricinus* defensins 8 were compared with selected closely related homologs of other hard tick species (**Supplementary Figure S1**). The signature residue for these types of defensin is the tyrosine next to the C-terminal cysteine residue, whereas the C-terminal motif for defensin 1 is CVMK (**Supplementary Figure S1**).

Another highly abundant transcript [Irseq_658490 (**Table 1**)] identified in the midgut of unfed ticks encodes an ortholog of *I. scapularis* Dae-2 (GenBank B7PLT0.2), which has recently been characterized as the effector molecule that protects the tick from infections with commensal bacteria on the host skin, e.g. Gram-positive *Staphylococcus epidermidis* (Hayes et al., 2020). The blastp search revealed the presence of a different, much less expressed form of Dae-2 (named Dae-2 type2) in the transcriptome of *I. ricinus*, encoded by the transcript Irseq_1462568 (**Table 1**). Orthologs of the dae-2 type2 form were only found in the closely related tick species *I. scapularis* and *I. persulcatus* (**Supplementary Figure S2**), but not in other hard ticks. While the protein (A0A131Y7K5) encoded by the Dae2 type1 transcript (Irseq_658490) was present in homogenates from unfed midgut, the Dae2 type2 protein was not detected, probably due to its low expression.

Several transcripts detected in the midgut of unfed *I. ricinus* were annotated as microplusins, as they encode histidine-rich proteins with a conserved pattern of cysteine residues to the characterized microplusin from the cattle tick *Rhipicephalus microplus* (Fogaca et al., 2004) or hebraein from *Amblyomma hebraeum* (Lai et al., 2004). These molecules are ubiquitously expressed in a variety of tick tissues and can be broadly categorized into two main groups based on their amino acid sequences: (i) type1 ricinusins and (ii) type2 microplusins (Urbanova et al., 2024). The group of ricinusins is represented by the transcript Irseq_818892 and microplusins by the much less expressed transcript Irseq_1035813 (**Table 1**). Orthologs of both types are found in the genome of *I. scapularis*, but with relatively low similarity to the original *R. microplus* microplusin (GenBank AAO48942.1) (**Supplementary Figure S3**). In line with their expression (TPM values), only the type1 ricinusin (A0A6B0UTF6) was detected in the unfed midgut by proteome analysis (**Table 1**).

Another dominantly expressed transcript (seqSigP-924189) in unfed midgut encodes an invertebrate c-type lysozyme (type1). On the other hand, expression of the related c-type lysozyme-like type2 (transcript Irseq_1030185) was marginal (**Table 1**). While distinct orthologs of the type1 lysozyme were found in a variety of hard tick species, the type2 ortholog was only identified in *I. scapularis* (**Supplementary Figure S4**). However, none of these lysozyme isoforms were detected at the protein level in the midgut of ticks (**Table 1**).

The *I. ricinus* transcript seqSigP-1078729 was annotated as acanthoscurrin, according to its identical *I. scapularis* ortholog acanthoscurrin 2-like (GenBank XP_029824929.2). However, despite the C-terminal glycine-rich motifs, the relationship with the characterized AMP from the tarantula spider (Lorenzini et al., 2003) is questionable (**Supplementary Figure S5**).

Several studies as well as our unpublished data from ticks indicate a possible role of GILT-related molecules in invertebrate immunity (Kongton et al., 2014; Zhou et al., 2017; Schleicher et al., 2018; Yang et al., 2020). The GILT transcript Irseq_909409 was very abundant in the midgut of unfed *I. ricinus* and its encoded protein (A0A0K8RMQ60) was clearly detected in midgut homogenates (**Table 1**). The orthologs of *I. ricinus* GILT with a well-conserved active site and GILT signature motifs are present in a variety of tick species, although their overall sequence similarity to other characterized invertebrate GILTs (Kongton et al., 2014; Zhou et al., 2017; Schleicher et al., 2018; Liu et al., 2019) is rather low (**Supplementary Figure S6**). In addition, the transcript seqSigP-678986, which encodes a GILT-related protein (V5HBD3) orthologous to the GILT-like protein 1 of *I. scapularis* (GenBank XP_042145453.1), was also highly abundant in the midgut transcriptome and proteome of unfed ticks (**Table 1**; **Supplementary Figure S6**).

### Antimicrobial activity of midgut defensins

**Figure 4:**
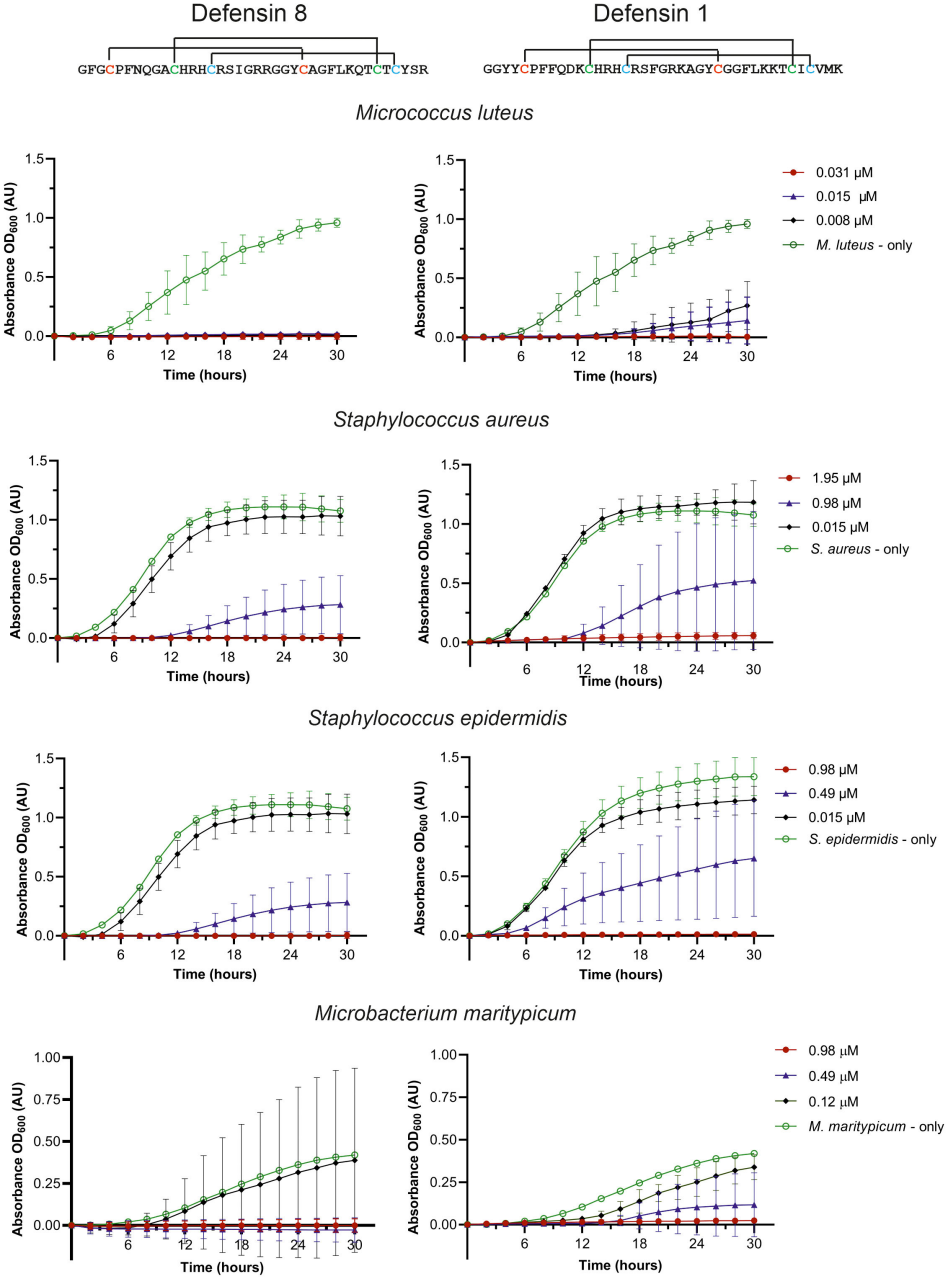
Effect of the synthetic mature defensin 1 and 8 on the inhibition of bacterial growth. Control bacterial growth curves (without defensins) are shown in green, at the minimum inhibitory concentration (MIC) needed for 100% inhibition in red, at the highest concentration with no inhibition in black, and at the concentration in the range of about 50% inhibition in blue.

We selected two midgut defensins to evaluate their potential as ‘guard’ AMPs. The antimicrobial activity of the synthetic mature defensins 1 and 8 was tested against four Gram-positive bacteria (*M. luteus, Staphylococcus aureus, S. epidermidis, Microbacterium maritypicum*) (**Figure 4**) and two Gram-negative bacterial strains (*Pseudomonas aeruginosa* and *E. coli*) (not shown). Both defensins inhibited the growth of all Gram-positive bacteria in a low concentration range of 0.008 µM to 1.95 µM; however, did not show any activity against the two Gram-negative bacteria at the highest concentration (250 µM) tested (**Table 2**).

**Table 2:** Bacterial growth inhibition assays by synthetic mature defensins and the effect of S-S bridges reduction by treatment with DTT.

Bacterial species	Minimum inhibitory concentration			
	Defensin 1 (mature)	Defensin 8 (mature)	Defensin 1 (mature) DTT – treated	Defensin 8 (mature) DTT – treated
***M. luteus***	0.031 µM (0.56 µg/mL)	0.008 µM (0.13 µg/mL)	3.0 µM (54 µg/mL)	0.006 µM (0.1 µg/mL)
***S. aureus***	1.95 µM (35 µg/mL)	1.95 µM (33 µg/mL)		
***S. epidermidis***	0.98 µM (18 µg/mL)	0.98 µM (16 µg/mL)		
***M. maritypicum***	0.98 µM (18 µg/mL)	0.49 µM (8 µg/mL)		
***E. coli***	>250 µM (>4500 µg/mL)	>250 µM (>4500 µg/mL)		
***P. aeruginosa***	>250 µM (>4500 µg/mL)	>250 µM (>4500 µg/mL)		

Since both peptides were highly effective in inhibiting *M. luteus* up to the lowest tested concentration (8 nM), the assay was repeated with lower concentrations. A minimum inhibitory activity (MIC) in the nanomolar range for the mature defensin 1 and the defensin 8, specifically 30 nM and 6 nM, respectively, was determined (**Table 2**). We also investigated the effect of disulfide bridge reduction on the MIC of defensins after their treatment with DTT. Reduction of the disulfide bridges significantly increased the MIC of the mature defensin 1 by ~100-fold (MIC of 3 μM), but had no effect on the activity of the mature defensin 8, which retained the same MIC of 6 nM (**Table 2**) in its oxidized form. Importantly, DTT alone had no effect on the growth of *M. luteus* at the highest concentration tested (500 μM).

## Discussion

Tick midgut is a primary site for interactions with ingested microbes, including pathogens that ticks transmit (Hajdusek et al., 2013). Despite the fact that microbes in the tick midgut environment appear to have optimal conditions for their proliferation, as the blood is nutrient-rich, extracellular proteases are absent, and the pH is neutral (Sojka et al., 2013), the opposite is true, and the midgut of several tick species has been described as close to sterile (Ross et al., 2018; Guizzo et al., 2020; Pavanelo et al., 2020; Maldonado-Ruiz et al., 2021). In the previous study, we have shown that both, Gram-positive (*M. luteus*) and Gram-negative (*Pantoea* sp.), bacteria are rapidly dramatically reduced in the midgut of unfed *I. ricinus* after experimental inoculation by the capillary feeding (Guizzo et al., 2022). This result suggests that this effective antibacterial response is crucial for maintaining low microbial levels in the tick gut. However, it was not clear whether the immediate response in the tick midgut is triggered rapidly or whether the tick has a constitutive guard immune system ready to eliminate the encountered bacteria. To investigate factors that may be responsible for this apparent refractoriness to bacterial proliferation, we analyzed the transcriptome of the midgut of unfed females challenged with either *M. luteus* or *Pantoea* sp. or sterile water by capillary feeding and compared it to that of midguts of unfed ticks that had not been treated at all. The transcriptome of ticks that ingested sterile water and bacteria-containing water drastically changed compared to that of unfed ticks; however, no clear upregulation of immune-related transcripts was observed. These results suggested that the antimicrobial factors responsible for the rapid bacterial clearance in the tick midgut, referred to as ‘guard’ AMPs, are expressed constitutively. Therefore, we focused our attention to the expression of transcripts encoding known AMPs (Kopacek et al., 2010; Hajdusek et al., 2013; Fogaça et al., 2021). Among the potential ‘guard’ AMPs, we identified the abundantly expressed defensin 8, which is annotated as the prepro-defensin 8 in the genome of *I. ricinus* (Cerqueira De Araujo et al., 2024). This defensin was not previously found in any tissue-specific transcriptome of *I. ricinus* (including salivary glands, hemocytes, fat body or midgut (Schwarz et al., 2013; Kotsyfakis et al., 2015; Perner et al., 2016, 2018; Urbanova et al., 2024). However, close orthologs of *I. ricinus* defensin 8 have already been described in other hard ticks, such as persulcatusin from the taiga tick *Ixodes persulcatus* (Saito et al., 2009), longicin from *Haemaphysalis longicornis* (Tsuji et al., 2007), and holosins from the Australian paralysis tick *Ixodes holocyclus* (Cabezas-Cruz et al., 2019)(**Supplementary Figure S1**). Proteomic analyses confirmed the presence of the mature defensin 8 in the midgut adult *I. ricinus* females (this study) as well as in nymphs in the early stages of their feeding (Kozelkova et al., 2023). The recently published longitudinal transcriptomic analysis of the midgut of *I. scapularis*, ranging from unfed to fully engorged and detached females (Lu et al., 2023) revealed the presence of several defensin isoforms (annotated as 4 kDa defensins or defensin-like isoforms), all closely related to *I. ricinus* defensin 8. The XP_040070918.2 isoform is abundantly expressed in unfed *I. scapularis* females with TPM values comparable to those of *I. ricinus* in this study (**Table 3**). Similar to *I. ricinus*, no transcript encoding an ortholog of defensin 1 was found in the *I. scapularis* midgut transcriptome (**Table 3**). Defensin 1 with the characteristic C-terminal motif CVMK is encoded by the duplicated genes prepro-defensin 1 and prepro-defensin 3 (Cerqueira De Araujo et al., 2024). The identical defensin molecule, designated def1 (GenBank AAP94724), was previously identified among the molecules induced in *I. ricinus* females by blood feeding (Rudenko et al., 2005). It was later reported to be expressed mainly in the tick midgut and active against Gram-positive bacteria (Chrudimska et al., 2011). In addition to the expression of the defensin 1 in the midgut of engorged *I. ricinus* females (Perner et al., 2016), an identical transcript was also identified in the transcriptome of the salivary glands of *I. ricinus* (Schwarz et al., 2013). Accordingly, the mature defensin-1 peptide was identified by the proteomics analyses in the midgut of nymphs (Kozelkova et al., 2023) and unfed females (this study) as well as in the salivary glands of *I. ricinus* (Bensaoud et al., 2022).

**Table 3:** Expression of “guard” genes orthologs in unfed *I.scapularis* midgut (from Lu et al., 2023).

Transcript	*I. scapularis* ortholog ID	UF1 TPM	UF2 TPM	UF3 TPM	Average TPM	Std Dev. TPM	UF *I. ricinus* average TPM^a^
**Defensin 8**	XP_040070918.2	2184,54	1847,72	2406,26	2146,17	281,24	2397
**Defensin 1**	nd	-	-	-	**-**	-	-
**Dae2-type1**	XP_040077735.1	1405,79	1240,27	1657,25	1434,44	209,96	3712
**Dae2-type2**	XP_040066081.1	4,26	5,75	1,92	3,98	1,93	71
**Microplusin/Ricinusin**	XP_040074415.1	25,51	40,29	45,5	37,10	10,37	448
**Microplusin - type 2**	XP_040074403.1	10,73	4,99	14,57	10,10	4,82	47
**c-type lysozyme 1**	XP_002399439.3	17,59	3,13	9,3	10,01	7,26	828
**Lysozyme-like**	XP_029833110.3	22,71	11,53	16,08	16,77	5,62	10
**Acanthoscurrin-like**	nd		–	–	**-**	–	27
**GILT**	XP_029827235.2	672,94	871,92	987,99	844,28	159,33	635
**GILT-like**	XP_042145453.1	143,07	140,79	195,19	159,68	30,77	1002

nd, not detected, ^a^values derived from the **Table 1** (this work).

Another putative ‘guard’ AMP of *I. ricinus* midgut is Dae-2 (Hayes et al., 2020). This antimicrobial effector molecule, first described in *I. scapularis* (Dae2*^Is^*), is present in the saliva and in the midgut and has been shown to eliminate Gram-positive bacteria such as *S. epidermidis* at a physiologically relevant concentration of 2 µM. The RNA interference (RNAi) silencing of Dae2*^Is^* significantly increased the load of *Staphylococcus* in comparison to that of the control. In addition, the injection of specific anti-Dae2*^Is^* antibodies via the anal pore increased the levels of *S. epidermidis* in the midgut and resulted in a significantly lower tick survival rate (Hayes et al., 2020). The *I. ricinus* transcript Irseq_658490, which encodes the ortholog of Dae2*^Is^* (Dae2 type1), was also abundantly expressed in the midgut of unfed ticks and is also present as a protein in midgut homogenates (**Table 1**). The transcripts of another form of Dae2 (Dae2 type2), identified in *I. ricinus*, and its ortholog in *I. scapularis* (**Supplementary Figure S2**) were only marginally expressed in the midgut of unfed ticks of both species (**Table 3**). Whether Dae2 type2 contributes to antibacterial defense remains to be investigated.

The antibacterial activity of cysteine- and histidine-rich proteins has only been demonstrated for two tick molecules; microplusin isolated from the hemolymph of the cattle tick *R. microplus* (Fogaca et al., 2004) and hebraein isolated from the hemolymph of the fed *A. hebraeum* females (Lai et al., 2004). *In vitro* studies have shown that the histidine residues of the microplusin of *R. microplus* chelate metallic ions such as copper and thus affect the respiration of *M. luteus* (Silva et al., 2009) and the fungus *Cryptococcus neoformans* (Silva et al., 2011). The gene expression of a microplusin transcript was upregulated by an experimental infection of the tick *Amblyomma aureolatum* with the tick-borne pathogen *Rickettsia rickettsii.* Knockdown of microplusin increased the acquisition of rickettsiae by feeding on infected rabbits, suggesting that this AMP plays an important role in bacterial control (Martins et al., 2019). There are two groups of microplusin-related molecules in *I. ricinus*: type 1, referred to as ricinusin, and type 2, which is more closely related to microplusin/hebraein due to the histidine-rich C-terminus (**Supplementary Figure S3**). The expression of type 1 ricinusin in the midgut of unfed *I. ricinus* was significantly higher than that of type 2 microplusin and, accordingly, only ricinusin was detected in the midgut proteome (**Table 1**). The TPM-based semi-quantitative expression profile of the corresponding orthologs in *I. scapularis* (Lu et al., 2023) also showed that the presence of type 2 microplusin is marginal compared to type 1 ricinusin (**Table 3**). Our previous analysis of the transcriptome of the fat body of *I. ricinus*, complemented by the proteome of the hemolymph (Urbanova et al., 2024), showed that ricinusin is one of the most abundant transcripts expressed in the fat body and that the corresponding protein is very abundant in the hemolymph. However, it remains to be experimentally proven whether the ricinusins and/or micropusins of *Ixodes* sp. play a bacteriostatic or fungistatic role in antimicrobial defense, as has been reported for microplusin of *R. microplus* (Fogaca et al., 2004).

C-type lysozyme is one of the very potent molecules active against Gram-positive bacteria, such as *M. luteus*, as we demonstrated two decades ago using lysozyme isolated from the midgut contents of the soft tick *Ornithodoros moubata* (Kopacek et al., 1999; Grunclova et al., 2003). Two immune-responsive c-type lysozymes were cloned and characterized from the EST library of the hemocytes of the hard tick *Dermacentor variabilis* and the embryonic cell line DAE100 of *Dermacentor andersoni* (Simser et al., 2004). Another c-type lysozyme called HlLysozyme was cloned from the hard tick *H. longicornis* and was shown to be expressed in different tick tissues (mainly in hemocytes) and to respond to the challenge with both Gram-positive and Gram-negative bacteria. Recombinant HlLysozyme was able to lyse Gram-positive *Micrococcus lysodeikticus* (*luteus*) in a concentration-dependent manner (Tanaka et al., 2010). The identification of a relatively abundantly expressed transcript encoding c- type lysozyme 1 in the midgut of unfed *I. ricinus* suggests its potential role in defense against Gram-positive bacteria. However, despite its high expression, the encoded enzyme was not detected by proteomic analysis (**Table 1**). The expression of orthologs of c- type lysozyme 1 and lysozyme-like type 2 in the midgut of *I. scapularis* (Lu et al., 2023) suggests their exclusive presence only in the unfed stage, with TPM values of both enzymes being rather low (**Table 3**). Therefore, the contribution of lysozymes to the total antimicrobial activity in the unfed midgut of *Ixodes* sp. is questionable.

GILTs belong to the family of thioredoxin-type oxidoreductases, which are responsible for the reduction of disulfide bonds in the acidic pH of lysosomes (Arunachalam et al., 2000; West and Cresswell, 2013). Three forms of GILTs have been identified in the fruit fly *D. melanogaster* (**Supplementary Figure S6**) and reported to be expressed mainly in the fat body and hemocytes (Kongton et al., 2014). Their knockdown in mutant flies increased infection by the Gram-negative *E. coli*, while overexpression decreased bacterial proliferation (Kongton et al., 2014). Conversely, CRISPR/Cas9 deletion of the gene encoding mosGILT of the mosquito *A. gambiae* resulted in refractoriness to malaria parasites *Plasmodium berghei* and *Plasmodium falciparum* (Yang et al., 2020). In addition, mutants had underdeveloped ovaries, egg development was reduced, and production of 20-hydroxyecdysone (20E) was lower (Yang et al., 2020). Indeed, RNA-seq analysis of mosGILT null mutants showed the modulation of transcripts related to the oogenesis and to the synthesis of 20-hydroxyecdysone (Arora et al., 2024). In addition, an upregulation of transcripts encoding anti-*Plasmodium* effectors, including one Clip domain serine protease (CLIPs), four thioester-containing protein 1 (TEP1), one peptidoglycan recognition proteins (PGRPs), and one defensin was observed in mutant mosquitos. The authors hypothesized that the reduction of the ovary development, which is a consequence of the absence of mosGILT, may intensify the immune response, reducing *Plasmodium burden* (Arora et al., 2024). In mosquito saliva, mosGILT binds to *Plasmodium* sporozoites, reducing the rate of cell crossing and parasite displacement, leading to a reduction in mouse infection (Schleicher et al., 2018). The role of GILTs expressed in the midgut of ticks remains to be investigated. In addition to their proposed role in midgut immunity, the disulfide-reducing activity of tick GILTs may also be involved in the activation of cysteine cathepsins in the acidic environment of the digestive endo-lysosomes (Sojka et al., 2013).

Functional *in vivo* testing of the antimicrobial activities of the identified ‘guard’ AMPs in the midgut of unfed ticks using RNAi-mediated silencing is difficult to impossible, as the RNAi machinery is significantly reduced in the metabolic resting state (De La Fuente et al., 2007). Therefore, only *in vitro* testing of either isolated and purified AMPs, such as microplusin from *R. microplus* (Fogaca et al., 2004) or lysozyme from *O. moubata* (Kopacek et al., 1999) or the production of recombinant proteins for antimicrobial testing, as demonstrated for Dae*^Is^* (Hayes et al., 2020), offer a solution for testing most ‘guard’ AMPs identified in this work. Defensins thus represent the only feasible exception for *in vitro* testing, as their size of about 40 amino acid residues allows their chemical synthesis. To test the antimicrobial activity of the mature defensin 1 and defensin 8, we used the experimental platform previously used to test other tick defensins (Tonk et al., 2014, 2015; Cabezas-Cruz et al., 2019). Our results clearly confirmed the antibacterial specificity of the two tested defensins 1 and 8 against Gram-positive bacteria *S. aureus*, *S. epidermidis* and *M. maritypicum* at typically reported micromolar concentrations (Saito et al., 2009; Chrudimska et al., 2011; Tonk et al., 2014, 2015). In contrast, the minimal inhibitory concentrations of defensins 1 and 8 against *M. luteus* were about two orders of magnitude lower and in the nanomolar range. As far as we know, such a low MIC has not been reported for any other invertebrate defensin. The chemically sophisticated synthesis of defensins made it possible to test role of disulfide bridges in the defensin antibacterial activity. Our results showed that the reduction of defensin 1 suppressed its activity against *M. luteus* by about 100-fold, whereas no change in MIC was observed for defensin 8 (**Table 2**). This difference suggests that disulfide bridges are important for defensin-1 activity, but not for the activity of defensin-8.

The expression of defensin 8 in the midgut of unfed *I. ricinus* and the expression of defensin 1 in the later stages of feeding (Perner et al., 2016), which show similar efficacy in inhibiting the growth of Gram-positive bacteria, lead us to speculate on the complementary roles of an ‘early’ and a ‘late’ defensin in the rapid elimination of Gram-positive environmental bacteria ingested during blood feeding. However, the detection of the ‘late’ defensin in the midgut proteome of unfed females, which may have been preserved from the nymphal stage (Kozelkova et al., 2023), suggest that the real situation is more complex.

Overall, our results indicate that the tick midgut deploys a number of non-induced immune factors as a first line of defense to protect ticks from environmental microorganisms during their long-term fasting period. Deciphering specific roles of the individual components of tick midgut immunity is a task for future studies.

## Data Availability

The datasets presented in this study can be found in online repositories. The names of the repository/repositories and accession number(s) can be found in the article/**Supplementary Material**.
