# Supplementary material for: The immune factors involved in the rapid clearance of bacteria from the midgut of the tick Ixodes ricinus
Source: Front Cell Infect Microbiol. 2024 Aug 13;14:1450353. doi: 10.3389/fcimb.2024.1450353 (PMC11347951; doi:10.3389/fcimb.2024.1450353)
Supplement: Supplementary file 1 [file DataSheet_1.docx]

**Supplementary file 1**

The hyperlinked Excel table with the filtered transcriptomic data containing 17,185 CDS from triplicate samples of midguts from unfed (UF1-3), water-fed (WF1-3), *Micrococcus luteus*-fed (MicF1-3) and *Pantoea* sp.-fed (PanF1-3) *Ixodes ricinus* females can be downloaded from the following link:

<https://proj-bip-prod-publicread.s3.amazonaws.com/transcriptome/Iricinus/InfectedMg/Supplementary_file_1.zip>
