# Supplementary material for: The immune factors involved in the rapid clearance of bacteria from the midgut of the tick Ixodes ricinus
Source: Front Cell Infect Microbiol. 2024 Aug 13;14:1450353. doi: 10.3389/fcimb.2024.1450353 (PMC11347951; doi:10.3389/fcimb.2024.1450353)
Supplement: Supplementary file 4 [file Image_2.pdf]

```
Ipersul(KAG0445185.1)        1   MEMRTVRIVCALLLGLITDLGEGGRGIPFVCDSPPSGIDWCLHVRDQKECTSVVKELCKRTVTVNGVPKVERLRSYTTEAWEPGVLVTDNCNSIPPMTAV 100
Iscap(XP_040066081.1)        1   MEMRTHHIVCALLLGLVTDLGECRRGVPFECNTPPSGIGWCLNEGDNKECTSVVKELCERTVTVNGERKVERLRSYTTQAWEPGVLVRDHCDSIPSMTAV 100   Dae2_type2
Irseq_1462568                1   --------------------------VPFECSTPPSGLRWCLNEGDDKECTSVVKELCERTVTVNGERKVERLRSYTTQAWEPGVLVRDHCDSIPSMTAV 74

Dae2_Iscap(XP_040077735.1)   1   --MKLFLISAALV-----VLGLAAVADAIGCSDPSPFQGRWVIGVDKKECVALVKEKCG------------NLRDYTTGRWVRGQHVKSNCGSIPKFTAI 81
Irseq_658490                 1   --MKLFLISAALV-----VLGLAAVSEAIGCSDPSPFQGRWVIGVDKKESVALVKEKCG------------NLRDYTTGRWVRGKHVKSNCGSIPKFTAI 81
Ipersul(KAG0421160.1)        1   --MKLFLISAALV-----VLGLAAVSDAIGCSDPSPFQGQWVIGVDKKECVALVKEKCG------------NLRDYTTGRWVRGKHVKSNCGSIPKFTAI 81   Dae2_type1
Rsanguin(XP_037511269.1)     1   --MKGYVVSAALL-----VLGMAVASQAIGCANPAPFKGKWVIGVDGKECVALVKEKCT------------GLRQYTTHSWRRGKHVRSNCGSIPRWSAI 81
Danders(XP_050044312.1)      1   --MQGSVITAALL-----VLGMAVASQAIGCANPAPFKGNWVIGVDGKECVALVKEKCT------------GLRQFTTHSWRRGKHVRSNCASVPRWSAI 81

Ipersul(KAG0445185.1)        101 ANFFGPGRSFDGSNYDNGHAAIFIRCLQGGEGGIE-------------------------------------------------------- 135
Iscap(XP_040066081.1)        101 ANFLGPGGSYDGSNQDNGHAAIVIRCLRGGEDGIEVYDQSRRNQLQRREMRHMSS-TRTSNGSTFYTIDIKSTATPLFDQDESSHPCRLSQ 190   Dae2_type2
Irseq_1462568                75  ANFFGPGRSYDGSNQDNGHAAIFIRCLRGGKDGIEVYDQSRRNPLKIREMEHMSS-TRNSNGSTFYTIDIKSTATPLFNQDESSHPCRRSQ 164

Dae2_Iscap(XP_040077735.1)   82  ATFLKPGNKYL------GHAAIFESCA---SDGIWVYDQWNAKPVERRKIRYGNTGK---------------------------------- 129
Irseq_658490                 82  ATFLKPGNKYL------GHAAIFESCA---SDGIWVYDQWNAKPVERRKIRYGNTGK---------------------------------- 129
Ipersul(KAG0421160.1)        82  ATFLKPGNKYL------GHAAIFESCA---SDGIWVYDQWNAKPVERRKIRYGNTGKPNYNGDNFYTIEV--------------------- 142   Dae2_type1
Rsanguin(XP_037511269.1)     82  ATFLD-GSKYR------GHAAIFESCA---SDGIWVYDQWNTAPVDRRKIRYGNS-KPNYNGDNFYMIEL--------------------- 140
Danders(XP_050044312.1)      82  ATFLD-GNKYR------GHAAIFESCA---SDGIWVYDQWNTSPVDRRKIRYGNS------------------------------------ 126
```

Supplementary Figure S2: Amino-acid sequence alignment of two types of domesticated amidase effector (Dae2). Irseq – *Ixodes ricinus* transcripts (this work); Ipersul – *Ixodes persulcatus*; Iscap – *Ixodes scapularis*; Rsanguin – *Rhipicephalus sanguineus*; Dander – *Dermacentor andersoni.* In brackets – GenBank Accession Nos.
