# Supplementary material for: The immune factors involved in the rapid clearance of bacteria from the midgut of the tick Ixodes ricinus
Source: Front Cell Infect Microbiol. 2024 Aug 13;14:1450353. doi: 10.3389/fcimb.2024.1450353 (PMC11347951; doi:10.3389/fcimb.2024.1450353)
Supplement: Supplementary file 5 [file Image_3.pdf]

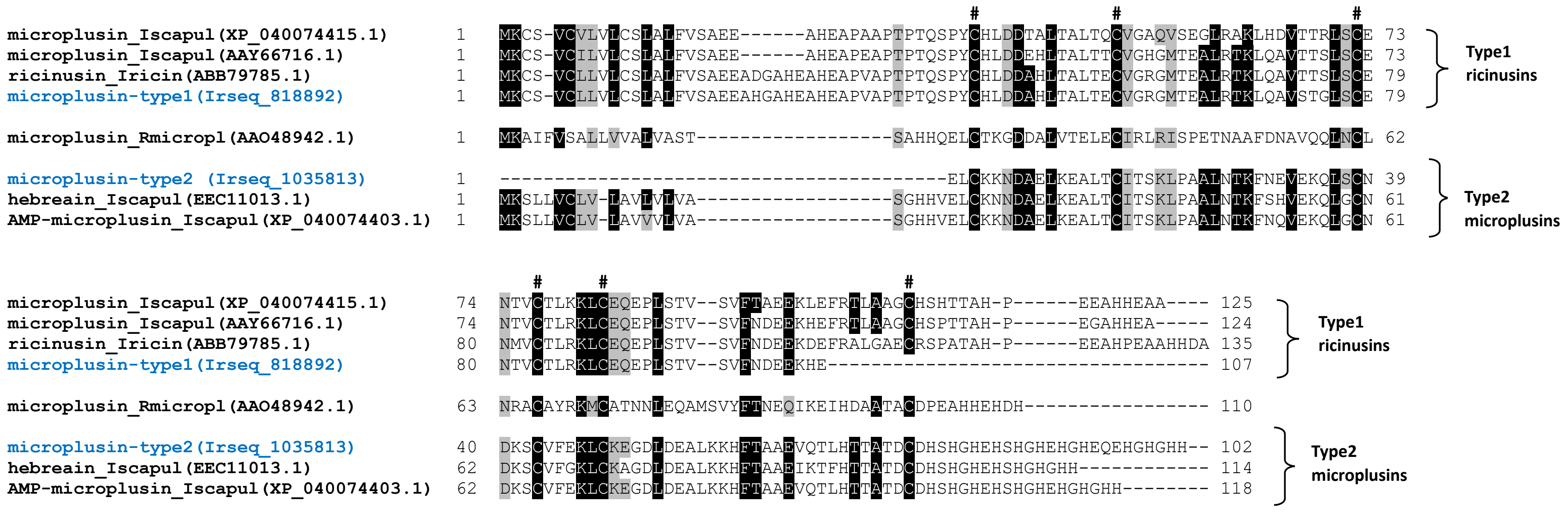

Supplementary Figure S3: Amino-acid sequence alignment of two types of microplusins identified in midguts of unfed *Ixodes ricinus.*
Irseq – *Ixodes ricinus* transcripts (this work); Iscap – *Ixodes scapularis*; Rmicropl – *Rhipicephalus microplus*; In brackets – GenBank Accession Nos. or transcripts identified in this work (in blue). Conserved cysteine residues are marked with hashtags.
