# Supplementary material for: The immune factors involved in the rapid clearance of bacteria from the midgut of the tick Ixodes ricinus
Source: Front Cell Infect Microbiol. 2024 Aug 13;14:1450353. doi: 10.3389/fcimb.2024.1450353 (PMC11347951; doi:10.3389/fcimb.2024.1450353)
Supplement: Supplementary file 6 [file Image_4.pdf]

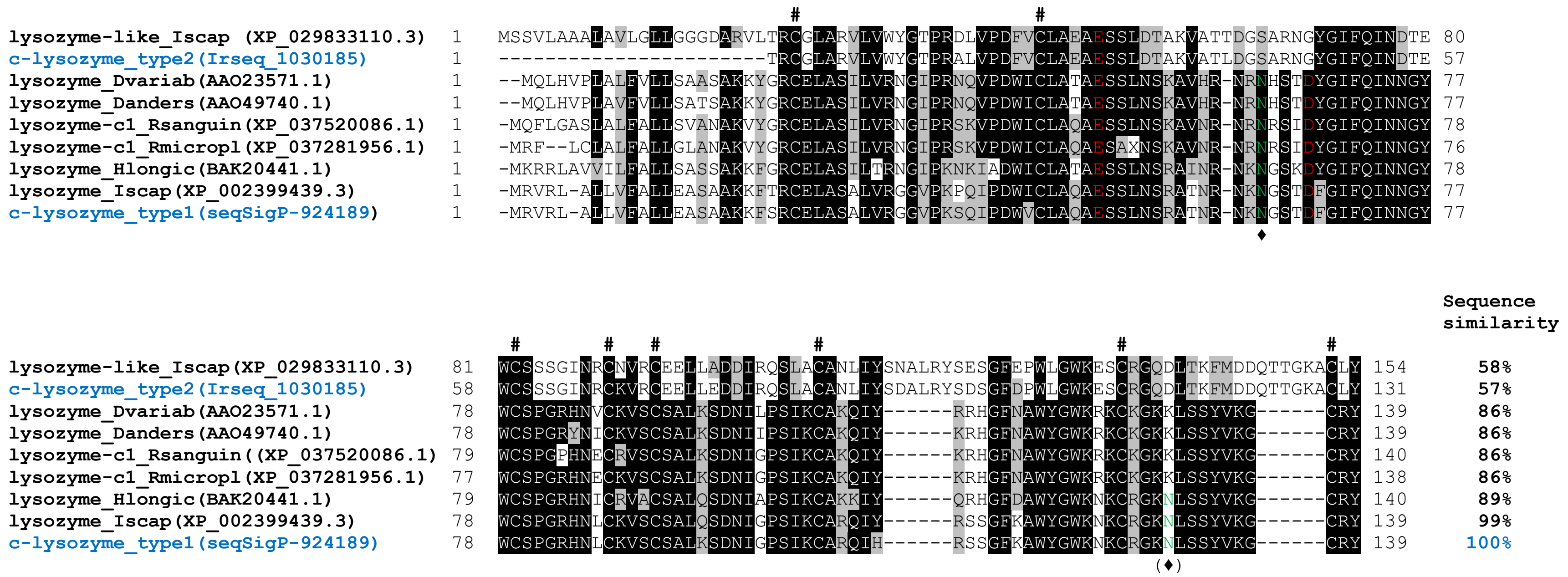

Supplementary Figure S4: Multiple amino-acid sequence alignment of selected tick lysozymes c. Irseq – *Ixodes ricinus* transcripts (this work); Iscap – *Ixodes scapularis*; Dvariab – *Dermacentor* variabilis; Danders – *Dermacentor andersoni*; Rsanguin – *Rhipicephalus sanguineus*; Rmicropl – *Rhipicephalus microplus*; Hlongic – *Haempahyslais longicornis*. In brackets – GenBank Accession Nos. or transcripts identified in this work (in blue). In red – active site residues, in green – potential N-glycosylation sites. Conserved cysteine residues are marked with hashtags.
