# Supplementary material for: The immune factors involved in the rapid clearance of bacteria from the midgut of the tick Ixodes ricinus
Source: Front Cell Infect Microbiol. 2024 Aug 13;14:1450353. doi: 10.3389/fcimb.2024.1450353 (PMC11347951; doi:10.3389/fcimb.2024.1450353)
Supplement: Supplementary file 8 [file Image_6.pdf]

```
                                                                                                   #
GILT-3_Dmelanog(NP_651165.1)       1  --MNKIFGLLFTLCLLL------------------VWP---TPGNGQSPDESRLLVAIHYEALCPDSMSFIRRRLYDALQDNDWWSVTDLKLYPFGKAGF 77
GILT-2_Dmelanog(NP_651166.1        1  ------MRAAVFVCLLL------------------GWVGVATPRRLRGPQADRLAITLYYEALCPYCMEFVTTQLNPSMVRQDRLPFTDLTLVPYGNAR- 75
mosGILT_Agambiae(AGAP004551)       1  ---M-----LFKSL---------------------LLLSV---YAVTCYGQSKVPVYVYYESLCPDSARFINEQLYPVAK--ELKKNLELHLVPFGKSSY 66
GILT-1_Dmelanog(NP_650287.3)       1  MSHK-----IAAVC---------------------LLMSC---LIATAYSAAKVPISIYYESLCPDSAKFITEQVYPAVKG-ELRDVVELTFVPFGKSQF 70
Iric_Irseq_909409                  1  MLPVILIGIIALLCVQA-----------------------------CEEPGAKVNVEVFYESYCPDSKRFVLEQLNSTYS--ELPNIMELQLIPYGKASR 69
GILT_Iscapul(XP_029827235.2)       1  MLPVILIGIIALLCVQA-----------------------------CEEPGAKVNVEVFYESYCPDSKRFVLEQLNSTYS--ELSNIMELQLIPYGKASR 69
GILT_Rsanguin(XP_049275333.1)      1  MLPVILIGIIALLCVQLLVIIFLMTSYGCS----LVG-LC--HPKTACVHDPKVSVEVFYETYCPDSRNFVLEQLNSTFA--ELPNIMQLQLVPYGKASR 91
GILT-like_Rmicropl(XP_037288490.1) 1  MLPVILIGIITLLCVQ------------------------------ACEHDPKVSVEVFYETYCPDSRNFVLEQLNSTFA--ELPNIMQLQLVPYGKASR 68
GILT-like_Dsilvar(XP_037556554.1)  1  MLPVILIGIIALLCVQ------------------------------ACEHDPKVSVEVFYETYCPDSRNFVLEQLNSTYA--ELPNIVQLQLVPYGKASR 68
GILT-like_Pclarkii(XP_045606082.1) 1  ------------------------------MRTSLLVLAG--LAAVLAQDPAPVKIDLYYETLCPYSIDFVTTQLYPTWT--ILSDIMEVELFPFGNANY 66

Iric_seqSigP-678986                1  ---MPRLDGLALLCIAVVICLVKSHETGRLFPEDLRWTNE--IDDNHLDAGRKVNVTLFHESMCPACKEFITNQLYTTYE--LLRDYMVVVLVPFGNGHI 93
GILT-like_Iscap(XP_042145453.1)    1  ---MTILDGLAALCLAVVICFAKSQEAGRLFPEDLRWTNE--IGDNHLDAGRKVNVTLFYESMCPYSREFITDQLYPTYE--LLRDYMVVVLVPFGNGHI 93
                                                                                                   CPDS-active site
                                                     #         #         #                                       #                         #                      #
GILT-3_Dmelanog(NP_651165.1)       78  YNN---TSTGESQVFCQHGVDECELNALHACIIETLD----------IRKAFNLIYCMLRSY------SNELGPCSRSMGV-DVSKARECKASRTTAEIL 157
GILT-2_Dmelanog(NP_651166.1)       75  -------TNDDGNVECQHGVMECELNAWHACILEHHD----------IAQSLKLIACMMRGK------KNRLEKCADHYQI-DVGDVKNCKKTRQVNDIL 151
mosGILT_Agambiae(AGAP004551)       67  TTQ-----GSDVMFTCHHGENECYGNKVHACAIQHIQGNSYQPNISKEDLTLDYVNCLMHRA-QLKDGAFPTKRCADEVKIDQWQAIMDCANSTEGSQLL 160
GILT-1_Dmelanog(NP_650287.3)       71  VTQ-----GSEVTFTCHHGPNECYGNKVHACAIEHIQANSYQVEYTRESLTMDFINCLMKAGKNFPDNVYPGQRCASENHINNWENIKTCANSTEGSVLL 165
Iric_Irseq_909409                  70  RQV---PSTKWYTFDCQHGDEECKGNLYQACAIHYHP---------DPSVHLPFIACMFES----SSPNSAYRRCALKSGF-DLEVLAKCNTDKEGNDLI 152
GILT_Iscapul(XP_029827235.2)       70  RQV---PSTKWYTFDCQHGDEECKGNLYQACAIQYHP---------DPSVHLPFIACMFES----SSPNSAYRRCALKSGF-DLEVLAKCNTDKEGNDLM 152
GILT_Rsanguin(XP_049275333.1)      92  REL---P-NKWYSFDCQHGDKECYGNLLQTCVIKYYP---------EPSQHLPLVVCMFSS----RNPNRAYESCAKKQGF-DTEALQKCVNGKEGNDLQ 173
GILT-like_Rmicropl(XP_037288490.1  69  REL---P-NKWYTFDCQHGDKECYGNLLQTCVIKYYP---------EPSQHLPLVVCMFSS----RNPNRAYESCAKKQGF-DLTALQKCISGKEGNDLQ 150
GILT-like_Dsilvar(XP_037556554.1)  69  REL---P-NKWYSFDCQHGDKECYGNLLQTCVIKYYP---------DPSQHLPLVVCMFSS----RNPNRAYESCAKKQGF-DLEALQKCVSGKEGNDLQ 150
GILT-like_Pclarkii(XP_045606082.1) 67  EQD-----GDGWTFTCQHGDGECHGNMIHACAKDHFK---------DINIEMEFVNCLLSA----DYPPNAGATCAAQVGQ-DWAPLDECVSSLEGQNLL 147

Iric_seqSigP-678986                94  DNKTLRNGKTYVSITCQHGVNECKGNKIEACAIKKYK---------MTSLWLPFVACMSRF----PDPHKRGKMCADSLHL-EWPAVGQCADGKEGQDLL 179
GILT-like_Iscap(XP_042145453.1)    94  DNKTLRNGKTYVSITCQHGVNECKGNKIEACAIKKYK---------MTSLWLPFVACMSRF----PDPHKQGKMCADSFHL-EWSAVGQCADGKEGQDLL 179
                                                       CQHGDxECxGNxxQxC - GILT signature motif
                                                                                         #                 #
GILT-3_Dmelanog(NP_651165.1)       158 APYGKETLKL----GISFVPTIVFENDFDPYDQRSIRNNFERHFCRQYLKKFNIKLPTCSAIL-------------------------------- 216
GILT-2_Dmelanog(NP_651166.1        152 RKYGKETAKV----SFQGVPAVALDNVYNADLSANLTDHFDAIFCAKYKEKFNKQLNNCQ----------------------------------- 207
mosGILT_Agambiae(AGAP004551)       161 KQHGDVTNKL--QSPLKSVPTVAFKQTYDDELQKLSVSSFRHALCKNLS-PQPVE---CLDLPSTGSAISSLGMIVT---VVAVLISRLL----- 241
GILT-1_Dmelanog(NP_650287.3)       166 RKAGESTMRL--KEPLTSVPTILFNEQFDKKVNDRAQVNLVGTICQYVSAPQPRI---CNQHN--GASTPSLASVSA---ILSSLLGLWFIRSFY 250
Iric_Irseq_909409                  153 VKYAEWTESVNAKKRLDFVPWIRMNSKDKM---FSAFTEFKKTLCEEYDRMAKAT---CPDATGNPQTQD-IPEACR--QVLPA----------- 227
GILT_Iscapul(XP_029827235.2)       153 VKYAEWTESVNAKKRLDFVPWIRMNSKDKM---FSAFTEFKKTLCEEYDRMAKTT---CPDATGNPQTQD-IPEACR--QVLPA----------- 227
GILT_Rsanguin(XP_049275333.1)      174 LRFAEWTESVNSKGRLEFVPWIRMNGATPG---GAFLP--------------------------------------------------------- 208
GILT-like_Rmicropl(XP_037288490.1) 151 LRFAEWTESVNSKGRLDFVPWIRMNGKDKM---YDAFTSFKRTLCEEYRSMVNTL---CTGDTPTTQPIE-IPQACK--VALS------------ 224
GILT-like_Dsilvar(XP_037556554.1)  151 LRFAEWTESVNSKGRLEFVPWIRMNGKDKM---YDAFTSFKRTLCEEYKSMVNTL---CTGDSPSTQPIE-LPQACK--VALS------------ 224
GILT-like_Pclarkii(XP_045606082.1) 148 HDVALQQEKL--DPTLYFVPWIIVNDVFNEDTVTDCQEDLKKVVCETYTGPTPDA---CASLKPTEPRSAHVNKAAAPRFAVRVSKR-----P-- 230

Iric_seqSigP-678986                180 YEMGRLTKDH--RPPIEYVPYIDFDGVHDQKAEDEARDNLLGVVCKKLGDVKPKV---CSGH--------------------------------- 236
GILT-like_Iscap(XP_042145453.1)    180 YQMGRLTEDH--RPPINYVPYIDFDGAHDQKAEDEARGNLLGVVCKKLGEVKPKV---CSGH--------------------------------- 236
```

Supplementary Figure S6: Multiple amino-acid sequence alignment of selected invertebrate gamma-interferon-inducible lysosomal thiol reductases (GILT) . Iric_– *Ixodes ricinus* transcripts (this work); Dmelanog – fruit fly *Drosophila melanogaster*; Agabmiae – malaria mosquito *Anopheles gambiae*; Pclarkii – red swamp crayfish *Procambarus clarkia*; Iscapul – *Ixodes scapularis*; Rsanguin – *Rhipicephalus sanguineus*; Rmicropl – *Rhipicephalus microplus*; Dsilvar – *Dermacentor silvarum* (all tick species in bold). In brackets – GenBank Accession Nos. In green – potential N-glycosylation sites. Conserved cysteine residues are marked with hashtags.
