# Supplementary material for: The immune factors involved in the rapid clearance of bacteria from the midgut of the tick Ixodes ricinus
Source: Front Cell Infect Microbiol. 2024 Aug 13;14:1450353. doi: 10.3389/fcimb.2024.1450353 (PMC11347951; doi:10.3389/fcimb.2024.1450353)
Supplement: Supplementary file 3 [file Image_1.pdf]

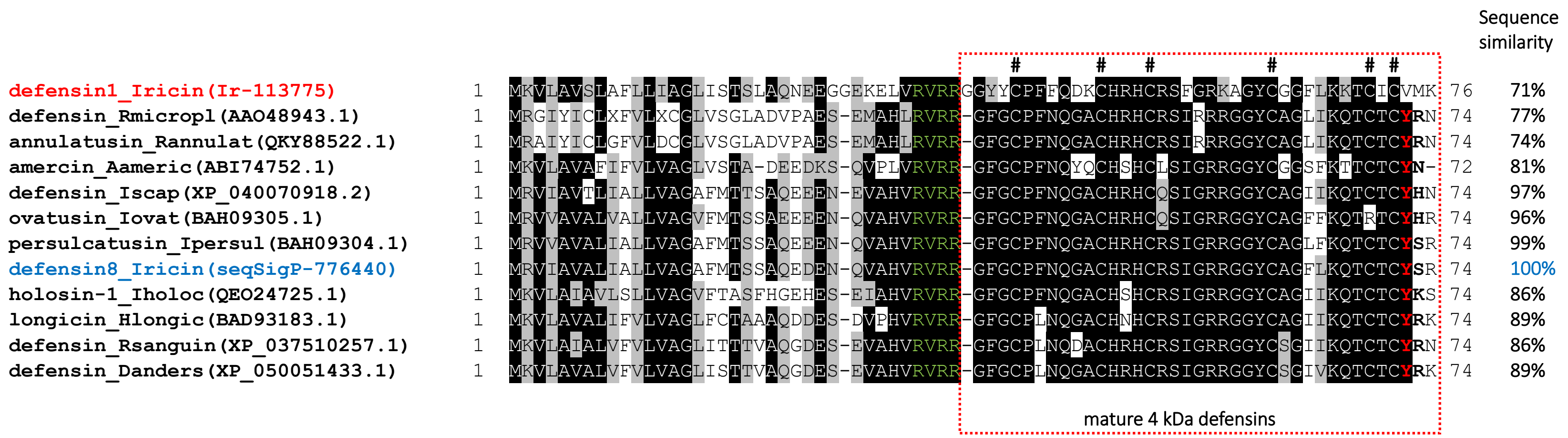

Supplementary Figure S1: Multiple amino-acid sequence alignment of selected prepro-defensins 8 orthologs. Iricin – *Ixodes ricinus*; Rmicropl – *Rhipicephalus microplus*; Rannulat – *Rhipicephalus annulatus*; Aameric – *Amblyomma americanum*; Iscap – *Ixodes scapularis*; Iovat – *Ixodes ovatus*; Ipersul – *Ixodes persulcatus*; Iholoc – *Ixodes holocyclus*; Hlongic – *Haemaphysalis longicornis*; Rsanguin – *Rhipicephalus sanguineus*; Danders – *Dermacentor andersoni*. In brackets – GenBank Accession Nos., defensin 8 (in blue] transcript ID from this work, defensin 1 (in red) transcript ID from Perner et al., 2016. RVRR (green) – furin cleavage site, bold red – signature tyrosine residue of defensin 8 orthologs. Conserved cysteine residues are marked with hashtags.
