# Supplementary material for: The immune factors involved in the rapid clearance of bacteria from the midgut of the tick Ixodes ricinus
Source: Front Cell Infect Microbiol. 2024 Aug 13;14:1450353. doi: 10.3389/fcimb.2024.1450353 (PMC11347951; doi:10.3389/fcimb.2024.1450353)
Supplement: Supplementary file 7 [file Image_5.pdf]

```
IriseqSigP-1078729        1   ---MKTLITSAIVLSLVLVCCQAHVVINGGRVGGGSYGCNCATLNFMTARRCCATVGTCCRLGGGLGLAGGLPLGGLGGYGGGLGGYGGGLGGYGPGLG- 96
acanthoscurrin-2_Iscapul  1   --MKTTLITSAIALSLVLVCCQAHVVINGGRVGGGSYGCNCATLNFMTARRCCATVGTCCRLGGGLGLAGGLPYGGLGGYGGGLGGYGGGLGGYGPGLG- 97
acanthoscurrin-2_Agomes   1   -----------------------DVYK-----------------------------------GGGGGRYGGGRYGGGGGYGGGLGGGGLGGGGLGGGKGL 42
acanthoscurrin-1_Agomes   1   MAFRMKLVVCIVLLSTLAVMSSADVYK-----------------------------------GGGGGRYGGGRYGGGGGYGGGLGGGGLGGGGLGGGKGL 65

IriseqSigP-1078729        96  ------------------------------------------------------------GYGGGLGGLGGGLGGYRGGLNVNLGGGFF-- 125
acanthoscurrin-2_Iscapul  97  ------------------------------------------------------------GYGG-------GLGGYRGGLNVNLGGGLF-- 119
acanthoscurrin-2_Agomes   43  GGGGLGGGGLGGGGLGGGGLGGGKGLGGGGLGGGGLGGGGLGGGGLGGGKGLGGGGLGGGGLGGGRGGY--GGGGYGGGYGGGYGGGKYKG 131
acanthoscurrin-1_Agomes   66  GGGGLGGGGLGGGGLGGGGLGGGKGLGGGGLGGGGLGGGGLGGGGLGGGKGLGGGGLGGGGLGGGRGGGYGGGGGYGGGYGGGYGGGKYKG 156
```

Supplementary Figure S5: Amino-acid sequence alignment of tick 'acanthoscurrins' with the characterized glycine-rich acanthoscurrins from the tarantula spider. Irseq – *Ixodes ricinus* transcript (this work); Iscap – *Ixodes scapularis* (GenBank XP_029824929.2); Agomes – tarantula spider *Acanthoscurria gomesiana* (GenBank CAD48603.1 and CAD48604.1 for acanthoscurrin 1 and 2, respectively).
